# The timing and duration of depressive symptoms from adolescence to young adulthood and young adults’ NEET status: the role of educational attainment

**DOI:** 10.1007/s00127-021-02142-5

**Published:** 2021-08-13

**Authors:** Karin Veldman, Sijmen A. Reijneveld, Johan Hviid Andersen, Trine Nøhr Winding, Merete Labriola, Thomas Lund, Ute Bültmann

**Affiliations:** 1 Department of Health Sciences, Community and Occupational Medicine, University Medical Center Groningen, University of Groningen, FA 10, P.O. Box 30001, 9700 RB Groningen, The Netherlands; 2 Danish Ramazzini Centre, Department of Occupational Medicine, University Research Clinic, Regional Hospital West Jutland, Herning, Denmark; 3 Section of Clinical Social Medicine and Rehabilitation, Department of Public Health, University of Aarhus, Aarhus C, Denmark; 4 NORCE Norwegian Research Centre, Bergen, Norway; 5 Center for Social Medicine, Bispebjerg and Frederiksberg Hospital, Copenhagen, Denmark; 6 Department of Public Health, University of Copenhagen, Copenhagen, Denmark

**Keywords:** Depressive symptoms, Educational attainment, NEET status, Adolescence, Young adulthood, Life course perspective

## Abstract

**Purpose:**

Depressive symptoms are negatively associated with labour market outcomes but whether the timing and duration of depressive symptoms or educational attainment (EA) affect NEET (Neither in Employment, Education, nor Training) is unknown. Therefore, this study aims to examine the effects of timing and duration of depressive symptoms in adolescence and the moderating and mediating role of EA on NEET in young adulthood.

**Methods:**

Data were used from 1512 participants in the Vestliv Study, a Danish prospective cohort study. Depressive symptoms were measured at age 14, 18 and 21. EA at age 21 and NEET at age 23 were derived from national registers. Logistic regression analyses and a 4-way decomposition approach were applied.

**Results:**

Among boys, depressive symptoms at ages 14 and 21 increased the risk of NEET (OR 1.65, 95% CI 1.00–2.74 and OR 2.20, 95% CI 1.37–3.53). Among girls, this regarded depressive symptoms at ages 18 and 21 (OR 1.76, 95% CI 1.26–2.46 and OR 1.59, 95% CI 1.13–2.22). For the duration of depressive symptoms, among boys any depressive symptoms increased the risk of NEET. Among girls, only persistent depressive symptoms increased the risk of NEET. EA did not mediate or moderate the association between depressive symptoms and NEET.

**Conclusion:**

The timing and duration of depressive symptoms in adolescence matter for the association with NEET in young adulthood, with a double burden for those with both depressive symptoms and low EA. The results emphasize the importance of support for those who experience depressive symptoms in the school-to-work transition.

**Supplementary Information:**

The online version contains supplementary material available at 10.1007/s00127-021-02142-5.

## Introduction

Depressive symptoms affect several labour market outcomes, including NEET status (Neither in Education, Employment nor Training) [1–14], which may be due to a negative effect of depressive symptoms on the level of energy, concentration and motivation [4]. Also, poor labour market participation may affect mental health negatively [6, 14]. To validly study the impact of depression on NEET, depression should be measured before young adults are exposed to the work environment [15]. A recent review of Clayborne et al. showed that adolescent depressive symptoms increase the risk of unemployment, but none of the included studied measured depressive symptoms at different time points [7]. Examining depressive symptoms at one time point, leaves it unclear whether the effect of depressive symptoms on NEET is dependent of the timing and duration of depressive symptoms.

With regard to the timing of depressive symptoms, the onset of depression early in life is a risk factor for more frequent and longer depressive episodes later in life [16], but evidence is scarce on how this translates to NEET status. Recently, De Groot et al. showed that the association between internalizing problems and having a paid work is independent of the timing of these problems, i.e. problems at all ages during (pre)adolescence were associated with having no paid job [17]. In line with this, Narusyte et al. found that internalising problems at different ages in childhood and adolescence were associated with sickness absence and receiving disability pension in young adulthood [18].

With regard to the duration of depressive symptoms, prior research has shown that a history of mental health problems is associated with poor labour market outcomes [19]. When a longer duration was compared to a shorter duration of mental health problems, an exposure–response relationship was found, i.e. the risk of having no paid work increased with the duration of internalizing problems [17].

It is known from the literature that depressive symptoms are associated with educational attainment [20–25], but evidence lacks almost fully on the role of educational attainment in linking depressive symptoms during adolescence to NEET status in young adulthood. Educational attainment might shape this association between depressive symptoms in adolescence and NEET status in young adulthood either as a moderator or as a mediator. As potential moderator, a higher level of educational attainment buffers the negative effect of depressive symptoms in adolescence on NEET status in young adulthood. Through education, young adults learn general skills, like solving problems and developing ideas, which add to their human capital [26]. Moreover, young adults have access to or are part of social networks through college attendance, which positively affects their social capital [26]. More human and social capital will help adolescents to cope better with their depressive symptoms [27, 28], and thus may buffer the effect of depressive symptoms on the risk of becoming in NEET. As potential mediator, depressive symptoms negatively affects educational attainment, and educational attainment affects labour market participation negatively, i.e., depressed adolescents are less likely to be successful at school, which is a disadvantage for entering the labour market [20–25]. The level of educational attainment determines chances at the labour market, e.g. a low level of educational attainment is linked to lower income and higher unemployment rates and a high level of educational attainment to higher income and lower unemployment rates [29]. A recent study of Johar et al. showed a mediating effect of the level of educational attainment on the association between adolescent depression and adult wages [9].

Given the limited evidence on the effects of timing and duration of depressive symptoms on NEET and the role of education, including the importance to inform policy and practice about when and how to intervene in the lives of adolescents and young adults, the present study was conducted. The aims of this longitudinal study were as follows: (1) to examine the effects of timing and duration of depressive symptoms from adolescence to young adulthood on NEET in young adulthood, and (2) to investigate whether the level of educational attainment moderates or mediates this association among 1512 Danish young adults participating in the Vestliv study. All analyses were conducted for the total sample and for boys and girls separately, as depressive symptoms among girls are more prevalent than among boys [30], and associations with educational attainment are found only among girls [22, 23].

## Materials and methods

### Ethical statement

The study was approved by the Danish Data Protection Agency, according to Danish law (Act on Research Ethics Review of Health Research Projects). Ethical approval or informed consent is not required for questionnaire and register-based studies. The reporting of this study conforms to the STROBE statement [31].

### Sample design

The West Jutland cohort study (Vestliv) is a prospective cohort study among Danish adolescents [32, 33]. All adolescents born in 1989 and living in the county of Ringkjøbing, Denmark in 2004 (*N* = 3681) were selected for participation in the Vestliv study. Contact information was derived from the Central Office of Civil Registration and from public schools. Participants were recruited either at school, or when not at school, by mail. A total of 3054 participants (83.0% of the initial sample, mean age 14.4 years, SD 0.49) completed the baseline questionnaire in 2004. In 2007, 2181 participants (71.4% of baseline, mean age 17.8 years, SD 0.38) and finally in 2010, 1945 completed the follow-up questionnaires (63.7% of baseline, participants were 20 or 21 years old, the exact age was not measured). The present study used 9-year follow-up data of 1512 Vestliv participants who had complete data for all waves.

#### Data management and linkage

Data on depressive symptoms was derived from the questionnaires. Data on educational attainment, NEET status, parental educational level and family composition was derived from Danish registers. NEET status was derived from the DREAM register, a Danish database that includes all social transfer payments, i.e. payments related to unemployment benefits, sickness absence compensation, disability pension, state educational grants, immigration and death [34]. Level of educational attainment of the participants and their parents was derived from the Registers of Education, which include individual-level information about enrolment and completion of Danish students per education institution [35]. Family composition was derived from the Danish Civil Registration System, which includes information of individual characteristics such as gender, date of birth, citizenship and identity of parents [36]. Data from the questionnaires and the registers was merged using the CPR number (a personal identification number) of each participant.

### Measures

Depressive symptoms were measured with the four-item version of the Center for Epidemiological Studies Depression scale for Children (CES-DC)[37] in 2004, 2007 and 2010 when participants were aged 14, 18 and 21 years, respectively. The four items were: ‘I was happy’, ‘I felt like friends were not friendly or that they didn’t want to be with me’, ‘I felt sad’ and ‘it was hard to get started doing things’. Participants could answer with ‘not at all’, ‘a little’, ‘some’ and ‘a lot’. The first item was reversed and higher CES-DC scores indicate higher levels of depressive symptoms. Cronbach’s alphas for the CES-DC were 0.63 in 2004 and 2007, and 0.70 in 2010, indicating an acceptable reliability. To measure timing, depressive symptoms were dichotomized for each time point at the 80th percentile (0 = No (severe) depressive symptoms, 1 = severe depressive symptoms) [38, 39]. To measure duration of depressive symptoms, dichotomized depressive symptoms were categorized across all time points as follows: (1) No exposure: participants who reported no depressive symptoms at any time point; (2) Single exposure: participants who reported depressive symptoms at one time point (3) Persistent exposure: participants who reported depressive symptoms at two or three time points.

NEET was defined as receiving social transfer payments in a 52-week period from week 35 in 2011 through week 35 in 2012, when participants were 23 years of age. Participants were categorized into two following groups: (1) in NEET when receiving social transfer payments, except maternity leave benefits and state educational grants and (2) not in NEET when receiving no social transfer payments, or when receiving maternity leave benefits or state educational grants (Lund et al. [40]). The social transfer payments of participants in NEET were either health-related benefits (sickness absence compensation, vocational rehabilitation benefits, and permanent disability benefits) or unemployment benefits, including leave benefits and training benefits offered to people after 4 weeks of unemployment.

Level of educational attainment concerned the educational level after compulsory school in October 2010 when participants were 21 years of age. The level of educational attainment was dichotomized as follows: (1) Completed secondary education: participants who had completed a secondary education, (2) Not completed secondary education: participants who were still attaining a secondary education, who dropped out of their last secondary education and never attained another, or who never attained a secondary education.

Parental educational level was based on years of education at the end of 2003. Parental educational level was classified as follows: (1) < 10 years, (2) 10–12 years, (3) 13–15 years, and (4) > 15 years.

Family composition was derived from the register at the end of 2003 and was dichotomized into the following: (0) Living with two parents or (1) living with one parent or not living with parents.

### Data analyses

Descriptive data were presented for all variables for the total sample, by sex and by NEET status. To test for differences between boys and girls, and between NEETs and non-NEETs, chi-square-tests were performed.

To assess the effect of timing and duration of depressive symptoms on NEET status, binary logistic regression analyses were performed. Crude associations between depressive symptoms and NEET status were calculated (Model 1), followed by adjustments for parental educational level, family composition and educational attainment (Model 2). For the assessment of the association between depressive symptoms at age 18 or 21 and NEET status, the analyses were adjusted for earlier depressive symptoms (i.e. at age 14 and/or age 18) (Model 3). Next, a 4-way decomposition approach was used to identify direct, mediation and interaction effects [41]. The total excess relative risk was decomposed into the following four components:

1. The effect of depressive symptoms on NEET status, while controlling for educational attainment, i.e. the Controlled Direct Effect (CDE).
2. The combined effect of depressive symptoms and educational attainment on NEET status, that is attributable to interaction but not to mediation, i.e. the Reference Interaction (INT_ref_).
3. The combined effect of depressive symptoms and educational attainment on NEET status, that is attributable to both interaction and mediation, i.e. the Mediated Interaction (INT_med_).
4. The effect of depressive symptoms on NEET statusthat is purely mediated by educational attainment, i.e. the Pure Indirect Effect (PIE).

In this 4-way decomposition approach  depressive symptoms at age 21 were not included, as educational attainment as potential mediator was measured at age 20/21, i.e. before depressive symptoms as determinant were measured. All analyses were conducted for the total sample and for boys and girls separately. Analyses were conducted in Stata version 16.

Characteristics of participants who dropped out or had missing data (*N* = 2169, 58.9%) were compared to characteristics of participants with complete data (*N* = 1512, 41.1%). Those characteristics include sex, parental educational level, family composition, level of educational attainment and NEET status.

## Results

### Sample characteristics

The total sample consisted of 1512 young adults (41.8% boys). Young adults who completed secondary education were more often in school or at work than young adults who did not (89.7% vs 80.7%). Young adults in NEET reported significantly more often severe depressive symptoms at age 14, 18 and 21, compared to young adults who were in school or at work. Table 1 shows the sample characteristics for the total sample, by sex and by NEET status at age 23.

**Table 1:** Background characteristics for the total sample, by sex and by NEET status at age 23 (*N* = 1512, Vestliv study)

Variables	Age^a^	Total	%	Boys	%	Girls	%	*p* value	NEET status				*p* value
		*N*		*N*		*N*			NEET		Not NEET		
									N	%	N	%	
Parental educational level	14							0.38					0.68
Low		126	8.3	48	7.6	78	8.9		25	7.8	101	8.5	
Medium/High		1386	91.7	584	92.4	802	91.1		297	92.2	1089	91.5	
Family composition	14							0.36					0.11
1 parent or no parent		114	7.5	43	6.8	71	8.1		31	9.6	83	7.0	
2 parents		1395	92.5	589	93.2	809	91.9		291	90.4	1107	93.0	
Educational attainment (*N*, %)	20/21							0.002					< 0.001
Not completed secondary education		185	12.2	97	15.3	88	10.0		62	19.3	123	10.3	
Completed secondary education		1327	87.8	535	84.7	792	90.0		260	80.7	1067	89.7	
Depressive symptoms	14							0.001					0.01
No (severe)		1211	79.4	529	83.7	672	76.4		240	74.5	961	80.8	
Severe		311	20.6	103	16.3	208	23.6		82	25.5	229	19.2	
Depressive symptoms	18							< 0.001					0.001
No (severe)		1190	78.7	541	85.6	649	73.8		230	71.4	960	80.7	
Severe		322	21.3	91	14.4	231	26.0		92	28.6	230	19.3	
Depressive symptoms	21							< 0.001					< 0.001
No (severe)		1166	77.1	519	82.1	647	73.5		219	68.0	947	79.6	
Severe		346	22.9	113	17.9	233	26.5		103	32.0	243	20.4	
Duration of depressive symptoms	14/18/21							< 0.001					< 0.001
No exposure		846	56.0	413	65.3	433	49.2		144	44.7	702	59.0	
Single exposure		410	27.1	147	23.2	263	29.9		98	30.4	312	26.2	
Persistent exposure		256	16.9	72	11.5	184	20.9		80	24.8	176	14.8	

^a^Age at which the variable was measured

### Timing and duration of depressive symptoms at ages 14, 18 and 21 and NEET status at age 23

For the timing of depressive symptoms, Table 2 shows that for the total sample, depressive symptoms at age 14, 18 or 21 increased the risk of being NEET at age 23 years [odds ratios (OR), 95% confidence intervals (CI), ranging from 1.43, 1.07–1.91 to 1.83, 1.39–2.41]. Among boys, severe depressive symptoms at age 14 or 21 increased the risk of being NEET [OR, 95% CI 1.65, 1.00–2.74; and 2.20, 1.37–3.53) (Table 3). Adjustment of parental educational level, family composition and educational attainment attenuated the association between depressive symptoms at age 14 and being NEET. Among girls, severe depressive symptoms at age 18 and 21 increased the risk of being NEET (OR 1.76, 1.26–2.46; 1.59, 1.13–2.22) (Table 4). Adjustment for earlier depressive symptoms revealed that only the effect of depressive symptoms at age 18 remained significant (OR 1.60, 1.13–2.27).

**Table 2:** Depressive symptoms at age 14, 18 or 21 and NEET status at age 23 for the total sample (*N* = 1512, Vestliv study)

	Model 1		*p* value	Model 2		*p* value	Model 3		*p* value^*^
	OR	95% CI		OR	95% CI		OR	95% CI	
Depressive symptoms (no-severe vs severe)									
Age 14	**1.43**	**1.07–1.91**	**0.02**	1.32	0.98–1.77	0.07			
Parental educational level (high vs low)				1.32	0.82–2.12	0.25			
Family composition (2 parents vs else)				1.34	0.85–2.09	0.21			
Educational attainment (completed vs not completed secondary education)				**2.16**	**1.53–3.04**	** < 0.001**			
Gender (girls vs boys)				**1.53**	**1.18–1.99**	**0.002**			
Depressive symptoms (no-severe vs severe)									
Age 18	**1.67**	**1.26–2.21**	** < 0.001**	**1.55**	**1.16–2.07**	**0.003**	**1.50**	**1.12–2.01**	**0.007**
Age 14							1.24	0.92–1.67	0.17
Parental educational level (high vs low)				1.35	0.84–2.17	0.22	1.36	0.84–2.19	0.21
Family composition (2 parents vs else)				1.38	0.88–2.16	0.16	1.36	0.86–2.12	0.19
Educational attainment (completed vs not completed secondary education)				**2.15**	**1.53–3.03**	** < 0.001**	**2.13**	**1.51–3.00**	** < 0.001**
Gender (girls vs boys)				**1.48**	**1.13–1.92**	**0.004**	**1.46**	**1.12–1.90**	**0.005**
Depressive symptoms (no-severe vs severe)									
Age 21	**1.83**	**1.39–2.41**	** < 0.001**	**1.65**	**1.24–2.18**	**0.001**	**1.50**	**1.12–2.00**	**0.007**
Age 18							**1.38**	**1.03–1.86**	**0.03**
Age 14							1.16	0.85–1.57	0.35
Parental educational level (high vs low)				1.38	0.86–2.22	0.19	1.41	0.87–2.27	0.16
Family composition (2 parents vs else)				1.36	0.87–2.12	0.18	1.35	0.86–2.12	0.19
Educational attainment (completed vs not completed secondary education)				**2.02**	**1.43–2.86**	** < 0.001**	**2.00**	**1.42–2.83**	** < 0.001**
Gender (girls vs boys)				**1.49**	**1.14–1.94**	**0.003**	**1.42**	**1.09–1.86**	**0.009**

Bold numbers refer to findings with a p-value < 0.05

Model 1: Crude, univariate, Model 2: Adjusted for parental educational level, family composition and educational attainment, Model 3: Model 2 + depressive symptoms at age 14 and/or age 18

*OR* odds ratio, *95% CI* 95% confidence interval

**Table 3:** Depressive symptoms at age 14, 18 or 21 and NEET status at age 23 for boys (*N* = 632, Vestliv study)

	Model 1		*p* value	Model 2		*p* value	Model 3		*p* value
	OR	95% CI		OR	95% CI		OR	95% CI	
Depressive symptoms (no-severe vs severe)									
Age 14	**1.65**	**1.00–2.74**	0.05	1.63	0.98–2.73	0.06			
Parental educational level (high vs low)				1.40	0.61–3.19	0.43			
Family composition (2 parents vs else)				1.54	0.73–3.26	0.26			
Educational attainment (completed vs not completed secondary education)				**2.20**	**1.33–3.65**	**0.002**			
Depressive symptoms (no-severe vs severe)									
Age 18	1.19	0.68–2.08	0.55	1.23	0.70–2.17	0.48	1.12	0.62–2.00	0.71
Age 14							1.60	0.95–2.71	0.08
Parental educational level (high vs low)				1.32	0.58–3.01	0.50	1.39	0.61–3.17	0.44
Family composition (2 parents vs else)				1.64	0.78–3.44	0.19	1.55	0.74–3.28	0.25
Educational attainment (completed vs not completed secondary education)				**2.21**	**1.34–3.66**	**0.002**	**2.21**	**1.33–3.66**	**0.002**
Depressive symptoms (no-severe vs severe)									
Age 21	**2.20**	**1.37–3.53**	**0.001**	**2.08**	**1.28–3.37**	**0.003**	**1.99**	**1.19–3.32**	**0.008**
Age 18							0.91	0.84–2.47	0.18
Age 14							1.44	0.49–1.67	0.76
Parental educational level (high vs low)				1.46	0.63–3.37	0.38	1.50	0.65–3.48	0.34
Family composition (2 parents vs else)				1.58	0.75–3.34	0.23	1.52	0.71–3.22	0.28
Educational attainment (completed vs not completed secondary education)				**2.07**	**1.24–3.44**	**0.005**	**2.08**	**1.25–3.47**	**0.005**

Bold numbers refer to findings with a p-value < 0.05

Model 1: Crude, univariate, Model 2: Adjusted for parental educational level, family composition and educational attainment, Model 3: Model 2 + depressive symptoms at age 14 and/or age 18

*OR* odds ratio, *95% CI* 95% confidence interval

**Table 4:** Depressive symptoms at age 14, 18 or 21 and NEET status at age 23 for girls (*N* = 880, Vestliv study)

	Model 1		*p* value	Model 2		*p* value	Model 3		*p* value
	OR	95% CI		OR	95% CI		OR	95% CI	
Depressive symptoms (no-severe vs severe)									
Age 14	1.27	0.89–1.81	0.19	1.20	0.84–1.71	0.33			
Parental educational level (high vs low)				1.30	0.73–2.31	0.38			
Family composition (2 parents vs else)				1.23	0.70–2.15	0.47			
Educational attainment (completed vs not completed secondary education)				**2.15**	**1.35–3.43**	**0.001**			
Depressive symptoms (no-severe vs severe)									
Age 18	**1.76**	**1.26–2.46**	**0.001**	**1.70**	**1.21–2.39**	**0.002**	**1.67**	**1.19–2.37**	**0.003**
Age 14							1.11	0.77–1.59	0.59
Parental educational level (high vs low)				1.38	0.77–2.48	0.28	1.38	0.77–2.48	0.28
Family composition (2 parents vs else)				1.25	0.72–2.20	0.43	1.25	0.71–2.18	0.44
Educational attainment (completed vs not completed secondary education)				**2.06**	**1.29–3.29**	**0.003**	**2.04**	**1.27–3.26**	**0.003**
Depressive symptoms (no-severe vs severe)									
Age 21	**1.59**	**1.13–2.22**	**0.007**	**1.47**	**1.04–2.07**	**0.03**	1.33	0.93–1.90	0.12
Age 18							**1.60**	**1.13–2.27**	**0.008**
Age 14							1.06	0.73–1.53	0.77
Parental educational level (high vs low)				1.35	0.75–2.41	0.32	1.42	0.79–2.54	0.25
Family composition (2 parents vs else)				1.24	0.71–2.17	0.44	1.25	0.71–2.19	0.44
Educational attainment (completed vs not completed secondary education)				**2.01**	**1.25–3.22**	**0.004**	**1.93**	**1.20–3.10**	**0.007**

Bold numbers refer to findings with a p-value < 0.05

Model 1: Crude, univariate, Model 2: Adjusted for parental educational level, family composition and educational attainment, Model 3: Model 2 + depressive symptoms at age 14 and/or age 18

*OR* odds ratio, *95% CI* 95% confidence interval

For the duration of depressive symptoms, any both a single or persistent exposure of severe depressive symptoms increased the risk of being NEET for the total sample and among boys (ORs ranging from 1.53 to 2.22) (Table 5). Among girls, only persistent exposure increased the risk of being NEET (OR 2.08, 1.41–3.06) (Table 5).

### Mediation and moderation effects of educational attainment on the association between depressive symptoms and NEET status

For the total sample and for boys and girls separately, on top of the effects of depressive symptoms, not completing secondary education increased the risk of being NEET (ORs ranging from 1.93 to 2.21, Tables 2,3, 4). This result indicated that for the models in which depressive symptoms increased the risk of being NEET, the effect of depressive symptoms and educational attainment on NEET status was multiplicative, as a logistic model is by nature.

**Table 5:** Duration of depressive symptoms from age 14–21 and NEET status at age 23 for the total sample and by sex (*N* = 1512, Vestliv study)

	Model 1		*p* value	Model 2		*p* value
	OR	95% CI		OR	95% CI	
Total						
Depressive symptoms						
No exposure (ref)	1			1		
Single exposure	**1.53**	**1.15–2.04**	**0.004**	**1.49**	**1.11–1.99**	**0.008**
Persistent exposure	**2.22**	**1.61–3.05**	** < 0.001**	**2.13**	**1.54–2.94**	** < 0.001**
Parental educational level (high vs low)				1.37	0.85–2.20	0.20
Family composition (2 parents vs else)				1.35	0.86–2.12	0.19
Educational attainment (completed vs not completed secondary education)				**1.37**	**1.15–1.63**	** < 0.001**
Boys (*n* = 632)						
Depressive symptoms						
No exposure (ref)	1			1		
Single exposure	**1.81**	**1.13–2.89**	**0.01**	**1.80**	**1.11–2.90**	**0.02**
Persistent exposure	**2.00**	**1.10–3.65**	**0.02**	**1.93**	**1.05–3.55**	**0.03**
Parental educational level (high vs low)				1.41	0.62–3.24	0.41
Family composition (2 parents vs else)				1.53	0.72–3.22	0.27
Educational attainment (completed vs not completed secondary education)				**1.40**	**1.08–1.82**	**0.01**
Girls (*n* = 880)						
Depressive symptoms						
No exposure (ref)	1			1		
Single exposure	1.32	0.91–1.90	0.14	1.24	0.85–1.80	0.27
Persistent exposure	**2.08**	**1.41–3.06**	** < 0.001**	**1.94**	**1.30–2.88**	**0.001**
Parental educational level (high vs low)				1.38	0.77–2.48	0.28
Family composition (2 parents vs else)				1.24	0.71–2.16	0.46
Educational attainment (completed vs not completed secondary education)				**1.42**	**1.12–1.81**	**0.004**

Bold numbers refer to findings with a p-value < 0.05

Model 1: crude, Model 2: adjusted for parental educational level, family composition and educational attainment

*OR* odds ratio, *95% CI* 95% confidence interval

For the total sample and for boys, the results showed that depressive symptoms were not associated with educational attainment (data not shown). Therefore, the 4-way decomposition approach was only performed for girls. Among girls, the 4-way decomposition approach did not reveal any moderating or mediating or interaction effects of the association between depressive symptoms and NEET status (Online resource Table S1).

Repeating the analyses with a CES-DC cut-off at the 90% percentile yielded similar but stronger associations. The comparative analyses showed that, compared to participants with complete data, those who dropped-out or had missing data, were more often boys (58.6% vs. 41.8%), more often had low educated parents (17.8% vs. 8.3%), more often came from single parent families (20.1% vs. 7.5%), more often had low levels of educational attainment (30.4% vs. 12.2%), and were more often NEET (26.8% vs. 21.3%) (Online resource Table S2).

## Discussion

In the present study, we found that the timing of severe depressive symptoms during adolescence matters for the risk of being NEET in young adulthood. Among boys, severe depressive symptoms at age 14 or 21 increased the risk of being NEET, whereas among girls, only severe depressive symptoms at age 18 were increased the risk of being NEET. Regarding the duration of severe depressive symptoms, we found that among boys any severe depressive symptoms increased the risk of being NEET. For girls, only persistent depressive symptoms increased the risk of being NEET. We found that both severe depressive symptoms, at ages 18 for girls and 14 and 21 for boys, and educational attainment increased the risk of being NEET. Educational attainment at age 20/21 did not moderate or mediate the association between depressive symptoms and NEET status.

For boys, severe depressive symptoms were dependent of the timing of these problems, but independent of the duration, with severe depressive symptoms at ages 14 and 21 and any duration increasing the risk of being NEET. This may be explained by the fact that boys and men tend to seek less help when feeling depressed than girls and women [42]. Compared to women, men may perceive more stigmas and more often believe that they have to deal with their feelings alone [43, 44]. Regarding the association of severe depressive symptoms at age 14 with NEET, a cautious interpretation is warranted as the results were borderline significant.

Among girls, only depressive symptoms at age 18 and a persistent exposure to severe depressive symptoms increased the risk of being NEET. An explanation may be that girls are especially susceptible for depressive symptoms during this phase in life, i.e. emerging adulthood. This is supported by the finding of an increased odds ratio for the association of depressive symptoms at age 21 and NEET status, although this association was no longer significant in the full adjusted model. Previous studies reported similar findings, i.e. that depressive symptoms in late adolescence and/or young adulthood are associated with work outcomes [2, 7–13], but only a few have examined the timing of depressive symptoms [17, 18]. In contrast to our findings, De Groot et al. [17] and Narusyte et al. [18] reported the effect of internalizing problems on having paid work, sickness absence and disability pension to be independent of the timing of these problems [17, 18], whereas we found that the effect of depressive symptoms on the risk of being NEET depended on timing but with different time points for boys and girls. This difference may be explained by the fact that De Groot et al. and Narusyte et al. did not stratify by sex, whereas we did. This explanation is supported by the fact that we also did not find an effect of the timing of depressive symptoms if we performed the analyses for our total sample, i.e. without stratification. More sex-stratified research taking depressive symptoms at different life phases into account is needed to improve our understanding of the effect of the timing of depressive symptoms with respect to later life work outcomes.

Depressive symptoms and educational attainment were found to have a multiplicative effect on NEET status, indicating that adolescents with severe depressive symptoms and poor educational attainment have a double burden. No moderating effects of the level of educational attainment were found, suggesting that the level educational attainment does not buffer adverse effects of depressive symptoms during adolescence on NEET status in young adulthood. It cannot be excluded that also other factors may be involved here, i.e. moderating effects might exist among adolescents coming from families with low socioeconomic position (SEP). For example, it was shown by Shafer et al. that children from families with low SEP have the highest health benefits when completing college [27]. Unfortunately, we were not able to conduct stratified analysis by family SEP because of the small number in each socioeconomic stratum.

We found no mediating effect of educational attainment on the association between depressive symptoms during adolescence and NEET status in young adulthood. Previous research of Johar et al. found for boys and girls that level of educational attainment mediated the association between depressive symptoms and employment wages, with larger mediation effects among girls than among boys [9]. Our contrasting findings might be due to the different outcome that we used, i.e. NEET status versus employment wages, or by the age of the study samples, i.e. Johar et al. measured employment wages at age 26–31 years, whereas we assessed NEET status at age 23. It may be that the mediating effect (and possibly also the moderating effect) of the level of educational attainment becomes more prominent later in adult life.

The present study has several strengths and limitations. We used data from a large population sample with 9 years of follow-up. With relatively high retention rates (almost 64% after 9-year follow up) and the use of register data (100% data on the level of educational attainment and on NEET status), the study had a very low loss to follow-up. Our sample size was *N* = 1512 after exclusion of participants who dropped out or had missing data. Comparison of participants with complete data with those who dropped out or had missing data suggests that selection may have occurred because of this, e.g. excluded participants reported lower (parental) educational levels, and were more often NEET. However, a previous comparison of those who participated and those who dropped out showed that differences in characteristics did not affect the relative risk estimates measured in the study [33], and selection bias of the risk estimates is unlikely. Next, the CES-DC was used to assess depressive symptoms at age 14, 18 and 21, and made full comparison of the level of depressive symptoms at the different ages possible. Officially, the CES-DC is used in the age range of 6–17 years. Differences between the child and adult version are minimal, and we chose to use the same measure over time to guarantee a full comparison. The Vestliv study has a multilevel sampling design, as participants were recruited at schools, and class level may have had an impact on the level of depressive symptoms. Data regarding school or class level were not available; therefore, we could not adjust in the analyses for potential effects of the multilevel design. However, depressive symptoms were measured when participants had already left the school, so at ages for which persistent class or school level effects on depressive symptoms are rather unlikely. NEET status was assessed only 2 years after educational attainment (at ages 23 and 21 years, respectively). Therefore, the effect of the level of educational attainment on NEET status may not have been completely visible yet and the results of this study could, therefore, be an underestimation of the real effect. The measure of NEET status used in this study is a rather crude measure, as it was defined as not receiving social transfer payments in a 52-week period. It may be that some young adults receive social benefits for a shorter period, for example, when young adults are between jobs or between education and employment, i.e. the associations may be underestimated.

The results of this study suggest that detection and early treatment of depressive symptoms during adolescence are important to improve young adults’ labour market outcomes. In Denmark, children or adolescents are not routinely screened on depressive symptoms [39], which makes it difficult to detect also less severe depressive symptoms. Early detection can initiate and target treatment to prevent depressive symptoms in adolescents and, on the long-term, may save health care costs. Given the long-term consequences and the enormous costs of depressive symptoms, there is a lot to gain on the individual and societal level. Smith and Smith have shown that the costs of childhood mental health problems in the US are immense: they reduce family incomes with 10,400 USD per year [45]. As this study with a life course perspective is one of the first on this topic, our findings have to be confirmed in further research, including a rigorous elaboration of the underlying mechanisms between depressive symptoms in adolescence and labour market participation in young adulthood to enhance the likelihood of a smooth transition into the labour market [46].

## Conclusion

In conclusion, we found that both the timing and duration of severe depressive symptoms during adolescence the risk for being NEET in young adulthood. Also, we found that depressive symptoms and educational attainment have a multiplicative effect on NEET status, but educational attainment did not moderate or mediate the effect of depressive symptoms on NEET status. Our findings show that much can be gained in participation in education or the labour market by tackling this major mental health problem issue. The results of this study show that (persistent) depressive symptoms in adolescence increase the risk of being NEET in young adulthood, suggesting the importance of treatment of depressive symptoms in adolescence.

## Supplementary Information

Below is the link to the electronic supplementary material.

Supplementary file1 (DOCX 17 KB)

## Data Availability

Upon request.

## Code availability

STATA version 16.1.
