# Supplementary material for: The timing and duration of depressive symptoms from adolescence to young adulthood and young adults’ NEET status: the role of educational attainment
Source: Soc Psychiatry Psychiatr Epidemiol. 2021 Aug 13;57(1):83–93. doi: 10.1007/s00127-021-02142-5 (PMC8761151; doi:10.1007/s00127-021-02142-5)
Supplement: Supplementary file 1 — Supplementary file1 (DOCX 17 KB) [file 127_2021_2142_MOESM1_ESM.docx]

Timing and duration of depressive symptoms from adolescence to young adulthood and young adults’ labour market participation: the role of educational attainment

*Social Psychiatry and Psychiatric Epidemiology*

Karin Veldman, PhD^1*^, Sijmen A. Reijneveld, MD, PhD^1^, Johan H. Andersen, MD, PhD^2^, Trine Nøhr Winding, PhD^2^, Merete Labriola, Phd^3,4^, Thomas Lund, PhD^5,6^, Ute Bültmann, PhD^1^

^1^ University Medical Center Groningen, University of Groningen, Department of Health Sciences, Community & Occupational Medicine.

^2^ Danish Ramazzini Centre, Department of Occupational Medicine – University Research Clinic, Regional Hospital West Jutland, Herning, Denmark

^3^ Section of Clinical Social Medicine and Rehabilitation, Department of Public Health, University of Aarhus, Aarhus C, Denmark.

^4^NORCE Norwegian Research Centre, Bergen, Norway

^5^ Center for Social Medicine, Bispebjerg & Frederiksberg Hospital, Copenhagen, Denmark

^6^ Department of Public Health, University of Copenhagen, Copenhagen, Denmark

* Corresponding author: Department of Health Sciences, Community & Occupational Medicine, University Medical Center Groningen, P.O. Box 30001, FA 10, 9700 RB, Groningen, the Netherlands. E-mail: [k.veldman@umcg.nl](mailto:k.veldman@umcg.nl).

Table S1. Total and decomposed effects of depressive symptoms on labour market participation due to mediation and/or interaction with educational attainment for girls (N=880, Vestliv study)

|  | Model 1 | | | Model 2 | | |
| --- | --- | --- | --- | --- | --- | --- |
|  | ERR | 95%CI | %  Attributable | ERR | 95%CI | %  Attributable |
| *Depressive symptoms*  *at age 14* |  |  |  |  |  |  |
|  |  |  |  |  |  |  |
| Total | 0.20 | -0.12;0.51 |  | 0.19 | -0.12;0.50 |  |
| CDE | 0.18 | -0.15;0.51 | 92.2% | 0.18 | -0.15;0.51 | 94.3% |
| INT_ref_ | -0.02 | -0.10;0.06 | -11.0% | -0.02 | -0.10;0.06 | -11.8% |
| INT_med_ | -0.02 | -0.09;0.05 | -9.3% | -0.02 | -0.08;0.05 | -9.3% |
| PIE | 0.05 | -0.03;0.11 | 28.1% | 0.05 | -0.01;0.11 | 26.7% |
|  |  |  |  |  |  |  |
| *Depressive symptoms*  *at age 18* |  |  |  |  |  |  |
| Total | **0.52** | **0.15;0.88** |  | **0.53** | **0.16;0.90** |  |
| CDE | **0.49** | **0.11;0.86** | 94.3% | **0.50** | **0.12;0.88** | 94.0% |
| INT_ref_ | -0.01 | -0.10;0.07 | -2.5% | -0.01 | 0.09;0.08 | -1.8% |
| INT_med_ | -0.02 | -0.08;0.06 | -2.1% | -0.01 | -0.08;0.06 | -1.5% |
| PIE | 0.05 | -0.01;0.11 | 10.3% | 0.05 | -0.01;0.11 | 9.3% |
|  |  |  |  |  |  |  |
|  |  |  |  |  |  |  |

ERR: Excess Relative Risk | CDE: Controlled Direct Effect | INT_ref_: Reference Interaction |

INT_med_: Mediated Interaction | PIE: Pure Indirect Effect

Model 1: crude | Model 2: adjusted for parental educational level and family composition

**Table S2** Characteristics of participants who dropped-out or had missing data versus participants with complete data

|  | **Dropped-out**  **(N=2169)** | | **Complete data**  **(N=1512)** | | **Effect size**  **(Cohen’s W)** | **p-value** |
| --- | --- | --- | --- | --- | --- | --- |
|  | **N** | **%** | **N** | **%** |  |  |
| Sex |  |  |  |  | 0.17 | <0.001 |
| Boys | 1272 | 58.6 | 632 | 41.8 |  |  |
| Girls | 897 | 41.4 | 880 | 58.2 |  |  |
| Parental educational level ^*^ |  |  |  |  | 0.14 | <0.001 |
| - Low | 370 | 17.9 | 126 | 8.3 |  |  |
| - Medium/High | 1700 | 82.1 | 1386 | 91.7 |  |  |
| Family composition |  |  |  |  | 0.17 | <0.001 |
| - 1 parent or no parent | 437 | 20.1 | 114 | 7.5 |  |  |
| - 2 parents | 1732 | 79.9 | 1398 | 92.5 |  |  |
| Educational attainment (N, %) |  |  |  |  | 0.21 | <0.001 |
| - Not completed secondary education | 660 | 30.4 | 185 | 12.2 |  |  |
| - Completed secondary education | 1509 | 69.6 | 1327 | 87.8 |  |  |
| NEET status |  |  |  |  | 0.06 | <0.001 |
| - NEET | 582 | 26.8 | 322 | 21.3 |  |  |
| - not NEET | 1587 | 73.2 | 1190 | 78.7 |  |  |

* Number of participants with information on parental educational level was 2070 for those who dropped out

^a^ Interpretation of Cohen’s W: Small 0.10 | Medium 0.30 | Large 0.50
